# In vitro grafting of hepatic spheroids and organoids on a microfluidic vascular bed

**DOI:** 10.1007/s10456-022-09842-9

**Published:** 2022-06-15

**Authors:** Flavio Bonanini, Dorota Kurek, Sara Previdi, Arnaud Nicolas, Delilah Hendriks, Sander de Ruiter, Marine Meyer, Maria Clapés Cabrer, Roelof Dinkelberg, Silvia Bonilla García, Bart Kramer, Thomas Olivier, Huili Hu, Carmen López-Iglesias, Frederik Schavemaker, Erik Walinga, Devanjali Dutta, Karla Queiroz, Karel Domansky, Bob Ronden, Jos Joore, Henriette L. Lanz, Peter J. Peters, Sebastiaan J. Trietsch, Hans Clevers, Paul Vulto

**Affiliations:** 1 Mimetas, Leiden, The Netherlands; 2 Oncode Institute, Hubrecht Institute, Royal Netherlands Academy of Arts and Sciences and University Medical Center, 3584 CT Utrecht, The Netherlands; 3 The Maastricht Multimodal Molecular Imaging Institute, Maastricht University, Maastricht, The Netherlands

**Keywords:** In vitro grafting, Vascularization, Liver organoids and spheroids, Microfluidics

## Abstract

With recent progress in modeling liver organogenesis and regeneration, the lack of vasculature is becoming the bottleneck in progressing our ability to model human hepatic tissues in vitro. Here, we introduce a platform for routine grafting of liver and other tissues on an in vitro grown microvascular bed. The platform consists of 64 microfluidic chips patterned underneath a 384-well microtiter plate. Each chip allows the formation of a microvascular bed between two main lateral vessels by inducing angiogenesis. Chips consist of an open-top microfluidic chamber, which enables addition of a target tissue by manual or robotic pipetting. Upon grafting a liver microtissue, the microvascular bed undergoes anastomosis, resulting in a stable, perfusable vascular network. Interactions with vasculature were found in spheroids and organoids upon 7 days of co-culture with space of Disse-like architecture in between hepatocytes and endothelium. Veno-occlusive disease was induced by azathioprine exposure, leading to impeded perfusion of the vascularized spheroid. The platform holds the potential to replace animals with an in vitro alternative for routine grafting of spheroids, organoids, or (patient-derived) explants.

**Supplementary Information:**

The online version contains supplementary material available at 10.1007/s10456-022-09842-9.

## Introduction

In the past few years, the ability to recapitulate features of human liver architecture and functionality in vitro has dramatically improved due to the use of three-dimensional culture methods. Spheroids and organoids are emerging as promising tools to study human organ development and regeneration, and can provide a model with enhanced translatability for drug response studies [1, 2]. One of the key limitations of current in vitro liver models is the lack of structured and functional sinusoidal vascular networks to ensure proper oxygenation and nutrition [3]. In fact, the liver is a highly vascularized organ, where specialized liver sinusoidal endothelial cells (LSECs) represent one of the predominant cell types, comprising about 70% of the non-parenchymal cell population [4, 5]. Studies have shown that all avascular organoids and spheroids with a size of 100 µm or larger are characterized by poor cell viability and functionality, which might translate into unstable and immature cultures [6, 7]. In addition, endothelial cells release angiocrine factors contributing to tissue growth, maturation, and functionality, as becomes apparent during organogenesis [8–10] and homeostasis [11, 12].

Various strategies have been pursued to introduce endothelial cells into liver microtissues. Vascularized liver spheroids were described in the context of liver cancer using a hanging-drop method to allow interaction between HepG2 and human umbilical vein endothelial cells (HUVECs) [13]. Subsequently, Inamori et al. demonstrated the possibility to vascularize primary hepatocyte spheroids by an endothelial cell encapsulation method [14]. Another approach included the generation of liver tissue using a layer-by-layer cell coating technique of hepatocytes, dermal fibroblasts, and HUVECs [15]. Others generated multicellular tissue constructs combining induced hepatic cells and HUVECs in decellularized liver scaffolds [16]. Although functional for studying cell–cell interaction, these methods did not yield stable, accessible, let-alone perfusable, microvascular networks. Alternatively, transplantation of spheroids/organoids into highly vascularized sites in mice allows vascularization of microtissues by the host [17] resulting in improved liver maturation and functionally [18, 19]. Although functionally powerful, xenografting has limited scalability and suffers from limited success rates and reproducibility. Moreover, the high cost, limited accessibility, and increasing ethical pressure limit the routine adoption of xenografting into laboratory practice [20].

Organ-on-a-chip technology is a promising tool to aid advanced tissue culture, as it can provide perfusion flow, gradients, and structured layering of tissues [21]. In a typical setup, two microfluidic channels are divided by a permeable membrane to separate the endothelial and hepatic compartments [22]. These chips have been shown to be advantageous for disease induction, liver zonation [23], and studying species-specific toxicity [24]. Direct interaction between a vascular network and a tissue of interest has been studied in a range of microfluidic setups, utilizing either vasculogenesis or angiogenesis approaches in hydrogel-supported cultures [25–27]. In order to accommodate the interaction of larger target tissues with microfluidic grown vasculature, so-called “open-top” chips were introduced [28]. For example, Oh et al. developed an open-top microfluidic chip in which microvasculature grown in a microfluidic network was allowed to interact with tumor cells placed on top of a porous membrane [29]. Nashimoto et al. utilized a micropillar approach to assess the angiogenic potential of mixed fibroblast-tumor spheroids, leading to juxtacrine interaction with vascular sprouts [30, 31]. Linn et al. developed a system of three communicating wells of a 384-well plate, in which mixed HepG2 spheroids, endothelial cells, and fibroblasts resulted in the self-organization of a perfusable 3D vascular network [32].

Notwithstanding this substantial progress, there is a need for a readily available platform that allows for routine vascularization of tissues in vitro. Here, we introduce a platform, dubbed the OrganoPlate Graft, comprising 64 microfluidic chips underneath a microtiter plate. Each chip allows for establishing a vascular bed through induction of angiogenesis from two main endothelial vessels. A target tissue is placed on top of the vascular bed for subsequent vascularization. We demonstrated vascularization of hepatic spheroids and organoids and perfusion of resulting microvascular networks. We further demonstrated the possibility to model veno-occlusive disease (VOD), an endothelial damage-associated disease in a liver context, upon exposure to azathioprine [33–37]. We foresee the use of the platform for in vitro grafting of a broad range of tissues, ultimately replacing animal grafting.

## Materials and methods

### Cell culture

Human primary RFP-labeled HUVECs (Alphabioregen, #RFP4) were cultured in T75 flasks (Corning, #734–2705) in MV2 (PromoCell, #C-22121) supplemented with 1% penicillin/streptomycin (Sigma, #P4333) and used at passage 4 till passage 8 for all experiments. Before use, a T75 flask was pre-coated with 50 µg/mL Purecol (Advanced BioMetrix, #5005-B) for 30 min at 37 °C in PBS (phosphate-buffered saline, Life tech #20,012,068) and then washed twice with sterile PBS. Cryopreserved Upcyte® Human Hepatocytes (UHH) from donor 653–03 were purchased from Upcyte technologies GmbH (Hamburg, Germany) and cultured as previously described [38]. Briefly, UHH were thawed in Thawing Medium (Upcyte technologies, #MHE001) seeded at a density of 10,000 cells/cm^2^ in collagen-type I-coated T75 flasks (Thermo, #156,499) and cultured in High-Performance Medium (HPM, Upcyte technologies, #MHE003). Media were replaced three times a week. All cells regularly tested negative for mycoplasma. For all cells, culture was performed in a humidified incubator at 37 °C and 5% CO_2_.

### Spheroids and organoids generation

To form hepatic spheroids, UHH at 100% confluency was dissociated using TrypLE™ Express Enzyme (Thermo, #12,604,021), and viability was assessed using the trypan blue dye exclusion method. 100 µl of HPM containing 20,000 UHH cells was transferred into each well of a Costar® ultra-low attachment 96-well plate (Corning, #CLS3474-24EA) and centrifuged for 1 min at 100 × *g*. For pre-vascularized spheroids, a mix of 1000 RFP-labeled HUVECs and 20,000 UHH were used. The plate was then placed in a humidified incubator (37 °C and 5% CO_2_). After 48 h, the spheroids were transferred onto the OrganoPlate Graft. Human hepatic organoids were generated from human fetal tissue as previously described [39]. After that, hepatic organoids were dissociated to single cells, and 6000 hepatocytes were added to each well of a Costar® ultra-low attachment 96-well plate in 100 µl of human Hep-Medium [39] and centrifuged for 5 min at 100 × *g*. After 3 days, organoids were transferred to the OrganoPlate Graft.

### Microfluidic cell culture

OrganoPlate culture was performed using the OrganoPlate Graft with 400 µm × 220 µm (w × h) channels (Mimetas BV, the Netherlands). Phaseguides had dimensions of 100 µm × 55 µm (w × h). Gel and perfusion channels had a length of 9 mm and 13 mm, respectively, with a downstream phaseguide wall interface of approximately 132 and 164 degrees. The phaseguide stability is expected to be around 200 Pa [40]. 2.5 µl of gel composed of 4 mg/ml Collagen I (AMSbio Cultrex 3D Collagen I Rat Tail, 5 mg/ml, #3447–020-01), 100 mM HEPES (Life Technologies, #15,630–122), and 3.7 mg/ml NaHCO_3_ (Sigma, #S5761) was dispensed in the gel inlet and incubated 15 min at 37 °C. Endothelial cells were detached by use of TrypLE™ Express Enzyme (Thermo, #12,604,021), counted and pelleted (5 min, 300 × g). The cells were applied to the system by seeding 2 µl of 1 × 10^7^ of cells/ml in the inlets of the perfusion channels. Subsequently, 50 µl of MV2 medium was added to the same inlets and the OrganoPlate was incubated in humified conditions at 37 °C and 5% CO_2_ for at least 1 h until cells attached on the bottom of the perfusion channels. After incubation, 50 µl medium was added to both channel outlets. The OrganoPlate was placed in the incubator (37 °C and 5% CO_2_) on an interval rocker switching between a + 14° and − 14° inclination every 8 min (OrganoFlow S, Mimetas) allowing bi-directional flow. Medium (50 µl each on inlet and outlet) was refreshed every 2–3 days. Endothelial tubes were cultured for 2–3 days before inducing microvascular bed formation.

### Microvascular bed formation

Sprouting of RFP-labeled HUVEC was induced by addition to the graft chambers of 50 µl of MV2 medium containing angiogenic factors: 50 ng/ml vascular endothelial growth factor (VEGF), 20 ng/ml basic fibroblast growth factor (bFGF), 2 ng/ml Phorbol 12-myristate 13-acetate (PMA), and 500 nM Sphingosine 1-phosphate (S1P). Stock solutions were prepared as follows: 100 μg/ml murine VEGF in MilliQ water (Preprotech, #100–20), 50 µg/ml bFGF in MilliQ water (Peprotech, #G00832 100-18B), 1 mM S1P (Sigma, #G00918) in 5% 1 M HCl, 95% DMSO, 10 μg/ml PMA (Sigma, # P1585) in 1% DMSO, and 100 μg/ml. Sprouting mix was refreshed every 2–3 days.

### Microvessels characterization

**Fig. 1:**
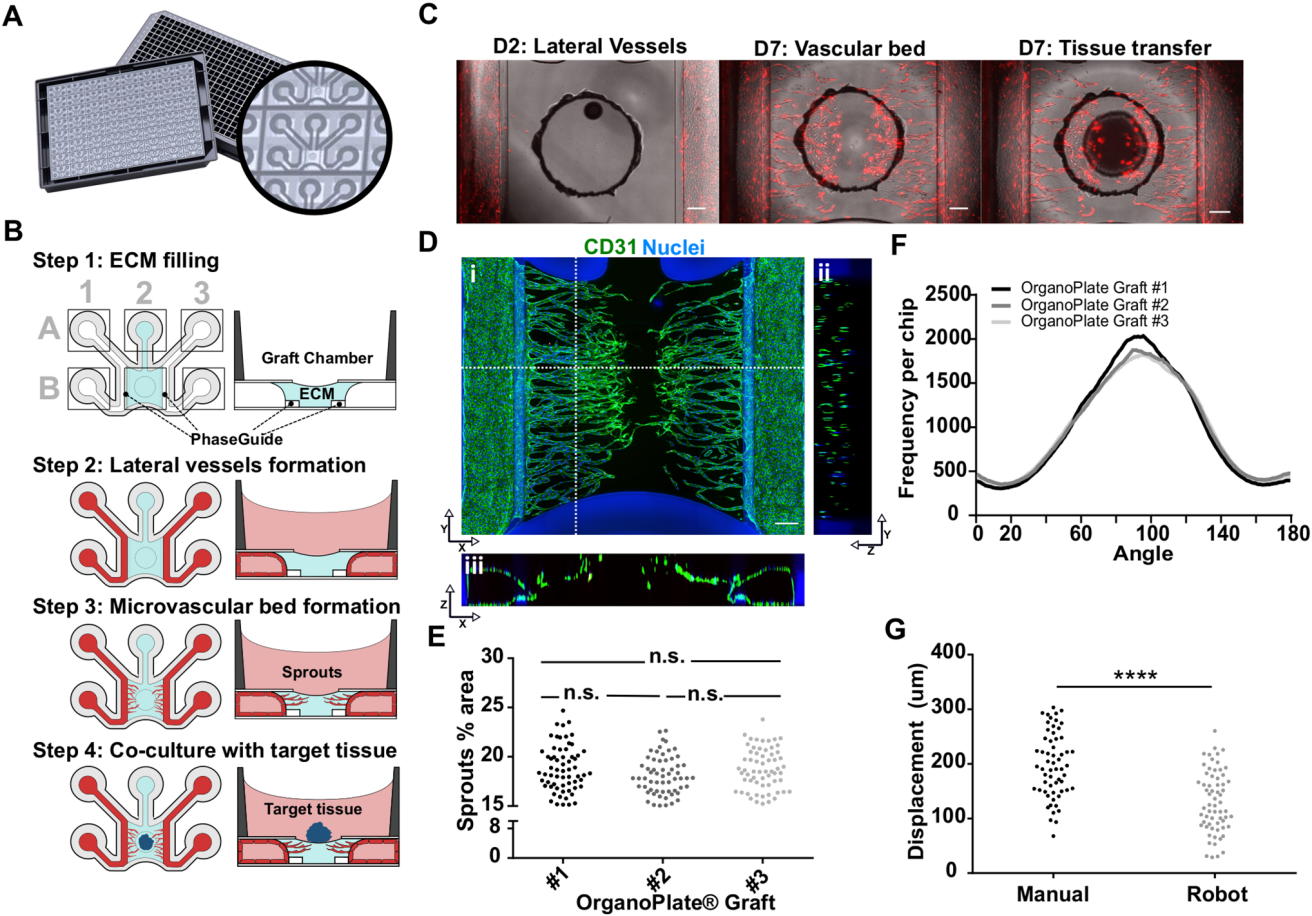
The OrganoPlate Graft allows for the generation of robust microvascular beds. **a** Top and bottom views of the OrganoPlate Graft with 64 microfluidic units positioned underneath a 384 microtiter plate. Each microfluidic unit makes use of a 2 × 3 array of wells from the microtiter plate (insert image). **b** Sequence of steps for generating a microvascular bed. Step 1: the graft chamber is filled with Collagen I gel (depicted in blue) through the gel inlet A2. Step 2: endothelial cells are seeded against collagen I gel (depicted in red) in the two lateral perfusion channels and form tubules upon application of perfusion flow. Step 3: Angiogenic factors are added to the graft chamber B2 to induce sprouting of the lateral vessels and formation of the vascular bed. Step 4: once microvessels have reached the opening on the graft chamber, a target microtissue is positioned on top of the microvascular bed to initiate interaction. **c** Images of an RFP-labeled HUVEC vascular bed formation prior to application of angiogenic factors (left) and after (middle). The target tissue is then positioned on the center of the graft chamber opening (right). Scale bar: 200 µm. **d** Maximum intensity projection (i) and cross sections (ii, iii) of a microvascular bed stained against CD31 (green) and nuclei (blue). Microvessels with a lumen are apparent. Location of cross sections is indicated by dash lines. Scale bars: 200 µm. **e** Quantification of sprout area coverage in 64 microfluidic units per OrganoPlate for three different plates (*n* = 192) after microvascular bed formation. Significance was calculated using one-way ANOVA and shown as non-significant (ns, *P* > 0.05). **f** Average distribution frequency of the orientation of microvascular structures (90 degrees indicates horizontal alignment) from 3 different OrganoPlate Grafts. **g** Evaluation of manual and robotic placement accuracy of target tissue on top of the microvascular bed. Statistical significance was attributed to values of *P* < 0.05 as determined by unpaired Student’s *t* test

To measure microvessels area and orientation, the microvascular bed was stained with Calcein AM green (Life Technologies, #C3099, 1:2000) in MV2 medium. After incubation for 30 min in the incubator (37 °C and 5% CO_2_) on an interval rocker switching between a + 14° and − 14° inclination every 8 min (OrganoFlow S, Mimetas), maximum intensity projections were acquired using the Micro XLS-C High Content Imaging Systems (Molecular Devices, US) at 4 × magnification. Maximum intensity projections from each chip were used to calculate the average microvessel orientation and microvessel area using the ‘orientationJ analysis’ tool and a modified version of the ‘morphology’ plugin in FIJI v.1.52. Data underlying Fig. 1f, e were obtained making use of automated image analysis routines that are described in supplementary Fig. 1c.

### Hepatic microtissue culture with microvessels

After 4–5 days of endothelial cell sprouting, hepatic microtissues were placed in the circular opening in the graft chamber on top of the microvascular bed. Before microtissue placement, medium was refreshed as follows: for hepatic spheroids, medium was aspirated from all graft chambers, in- and outlets and replaced with 50 µl MV2 (in- and outlets) and 50 µl HPM (graft chamber); for hepatic organoids, medium was aspirated from all graft chambers, in- and outlets and replaced with 50 µl Hep-Medium (in- and outlets). Graft chamber was left dry to allow overlay of organoids with Matrigel.

For manual placement of hepatic spheroids, microtissues were picked up from the ultra-low attachment plate together with 20 µl culture medium using wide-bore tips (Pure™ 200G Pipet Tips, VWR, #53,225–682). The pipette tip was then placed above the circular opening of the graft chamber allowing the spheroid to slowly sediment from the pipette tip onto the microvascular bed. For placement with liquid handler, the same procedure was followed using the OT-2 robot (Opentrons). Position accuracy of the spheroids for both manual and automated placement was determined by measuring the relative position along XY axis versus the center of the graft chamber using FIJI v.1.52 and translating to a radial offset. The OrganoPlates were then placed in the incubator (37 °C and 5% CO_2_) and were kept static for 1 h to allow the spheroids to adhere to the underlying collagen-I matrix. This prevents displacement during initial stages of rocking as well as during subsequent medium changes.

Hepatic organoids were transferred manually using a standard p10 micropipette into the circular opening in the graft chamber. After placement, 5 µl Matrigel GFR (Corning, #356,231) was added on top of each organoid, and the gel was allowed to polymerize for 10 min at 37 °C. At the end of the incubation, 50 µl Hep-Medium was added to all graft chambers.

The OrganoPlates were then placed on an interval rocker switching between a + 14° and − 14° inclination every 8 min (OrganoFlow S, Mimetas) allowing bi-directional flow. Media were refreshed every 2–3 days.

### Quantification of vessel intensity over time

Fluorescent images were acquired every 2–3 days using an ImageXpress XLS Micro High content imaging system at 37 °C (4 × objective). FIJI v.1.52 was used to quantify the relative RFP-HUVEC vessel intensity by drawing a rectangular selection excluding the lateral perfusion channels and measuring the mean signal intensity.

### Barrier integrity assay

Barrier integrity of lateral vessels was examined by addition of MV2 media containing 0.5 mg/ml FITC-dextran (150 kDa, Sigma #46,946) to both perfusion channels (40 µl in the inlets and 20 µl in the outlets). 20 µl of fresh media without dye was added to the graft chamber. Images were acquired every 15 s for 8 min using an ImageXpress XLS Micro High content imaging system at 37 °C (4 × objective).

### Immunohistochemistry

For direct immunostaining in the OrganoPlate Graft of co-cultures, cells were fixed with 3.7% formaldehyde (Sigma, #252,549) in PBS (phosphate-buffered saline, Life tech #20,012,068) for 15 min. After washing twice for 5 min with PBS, permeabilization and blocking were carried out together using 0.3% Triton X-100 (Sigma, #T8787), 2% FCS, 2% bovine serum albumin (BSA) (Sigma, #A2153), and 0.1% Tween 20 (Sigma, #P9416) in PBS for 2 h. Subsequently, cells were incubated with primary antibodies overnight, washed three times, incubated with secondary antibodies for 2 h, and washed three times with 4% FCS in PBS. The following antibodies were used for immunohistochemistry: Mouse a-CD31 (Dako, M0823, Clone JC70A, 1:20), Goat a-Albumin FITC conjugated (Bethyl, A80-229F, 1:200), Mouse a-HNF4A (Thermo Fisher, MA1199, 1:500), Mouse a-MRP2 (Abcam, M2 III-6, ab3373, 1:100), Mouse a-acetylated tubulin (Sigma, T6793, 1:4000) Mouse a-E-cadherin (BD, 610,181, 1:300), and Goat a-Mouse AlexaFluor 647 (Thermo Fisher, #A31571, 1:250). After nuclear stain with Hoechst (Thermo Fisher Scientific, #H3570, 1:2000 in PBS) cells were stored in PBS (50 µl in all graft chambers and in- and outlets). Tissue clearing was performed using CUBIC reagents (TCI Chemicals, Cubic-L, and CUBIC-R +) according to manufacturer instructions.

All steps were performed at room temperature (RT) and under the presence of flow. Cells were imaged with the EVOS FL2 Auto, ImageXpress Micro XLS, and Micro XLS-C High Content Imaging Systems (Molecular Devices, US).

For immunostaining of hepatic spheroids sections, vascularized spheroids were first removed from the graft chamber at different timepoints of the co-culture, transferred to cryomolds, overlaid with Cryo-Gel (Leica, #39,475,237) and frozen by floating in an ethanol/dry ice bath. After solidification, cryomolds were stored at − 80 °C until cryosectioning. Cryosectioning was performed using a cryostat (Cryotome® FE, Thermo Scientific) at − 20 °C. 10 μm cryosections were collected on SuperFrost® Plus slides (Fisher Scientific, #10,149,870). The slides were air-dried and fixed with 3.7% formaldehyde (Sigma, #252,549) in PBS for 15 min. After washing twice for 5 min with PBS, sections were permeabilized with 0.5% Tween in PBS for 10 min and blocked with 2% BSA and 0.05% Tween in PBS. Sections were incubated with Mouse a-CD31 (Dako, M0823, Clone JC70A, 1:20), Goat a-Albumin FITC conjugated (Bethyl, A80-229F, 1:200) for 2 h, washed three times 5 min with washing solution (0.05% Tween in PBS) and incubated with secondary antibody for 1 h. After washing three times, nuclei were stained with DAPI (Thermo Fisher Scientific, #R37606). Sections were mounted with Vectashield (Vector Laboratories, #H-1400) and imaged with EVOS FL2 Auto.

#### Perfusion assay

Interconnection and perfusion between the left and right side of the vascular network in the presence of microtissues were determined by addition of 100 µl MV2 medium containing 0.25 mg/ml FITC-Dextran (150 kDa, Sigma #46,946) to the left perfusion inlet, while 50 µl of MV2 medium was added to the left perfusion outlet and right in- and outlets. Medium in the Perfusion channels was removed for the course of the assay. Images were acquired every 60 s for 5 min using an ImageXpress XLS Micro High content imaging system at 37 °C (4 × objective). Perfusability of the vascular network was identified, when perfusion with fluorescent dye was observed via the lumen of vascular network from the left channel to the right perfusion channel. Perfusable chips were identified manually, when apparent flow of the dye was observed to traverse the vascular network reaching the right perfusion channel.

#### Transmission electron microscopy

A co-culture of hepatic organoids with vascular bed was fixed with 1.6% glutaraldehyde for 24 h at 4 °C and removed from the OrganoPlate. Then, samples were kept in wash buffer (0.1 M cacodylate) until further processing. Samples underwent an additional fixation step with 1% osmium tetroxide and 1.5% potassium ferricyanide in wash buffer for 1 h in the dark at 4 °C. Further, the samples were progressively dehydrated in ethanol 70–100% and infiltrated with Epon resin for 2 days. Then, samples were embedded in Epon resin, which polymerized for 2 days at 60 °C. Ultrathin sections were cut using an ultramicrotome (Leica Ultracut UCT) and mounted on Formvar-coated copper grids. Sections were stained with 2% uranyl acetate in water and lead citrate. Sections were imaged using a Tecnai T12 electron microscope and an Eagle 4 k*4 k CCD camera (ThermoFisher Scientific).

#### Bile canaliculi staining

Staining with CDFDA was performed to visualize active bile canaliculi in the hepatic organoids co-cultured with microvessels. Briefly, a stock solution containing 5 mM 5-(and-6)-carboxy-2′,7′-dichloro-fluorescein diacetate (CDFDA) (Sigma-Aldrich, #2188) was prepared in 100% Dimethyl Sulfoxide (DMSO) (Sigma, #D8418), aliquoted, and stored at -20 °C. For the assay, culturing media were aspirated in all graft chambers and perfusion in- and outlets and 50 µL of Hep-Medium containing 5 µM CDFDA (1:1000), and Hoechst (Thermo Fisher Scientific, #H3570, 1:2000 in PBS) was added to each well. The OrganoPlate Graft was incubated for 30 min in the incubator (37 °C, 5%, CO2) on an interval rocker switching between a + 14° and − 14° inclination every 8 min (OrganoFlow S, Mimetas). As negative control, Hep-Medium media containing 0.1% DMSO and Hoechst (Thermo Fisher Scientific, #H3570, 1:2000 in PBS) was prepared. At the end of the incubation, chips were washed 6 times with PBS and imaged Micro XLS-C High Content Imaging Systems (Molecular Devices, US).

#### Functional analysis of hepatic spheroids and organoids

To determine the levels of albumin secreted over time by spheroids and organoids, co-culture media were collected from perfusion channels and graft chambers (day 3, 5, and 7 for spheroids, day 7, 14, and 21 for organoids) and analyzed using the Human Albumin ELISA Quantification kit (Bethyl Laboratories Inc., E80-129, sample dilution 1:125).

Urea production was determined by a colorimetric assay kit (Biovision, #K375-100, sample dilution 1:5) following the manufacturer’s protocol.

#### Viability determination

For this experiment, vascular beds were formed using HUVECs (Lonza, #2519AS) using a modified concentration of S1P (250 nM) and PMA (10 ng/ml), and endothelial cells were sprouted for 3 days. Spheroids were composed of 7500 UHH from donor 151–03. AZA was administered in both, perfusion lanes and graft chamber at a concentration range from 0.66 µM to 160 µM for 72 h (days 2–5 of co-culture with microvessels). CellTiter Glo3D (Promega, #G9681) was used to determine the toxic effect of Azathioprine on liver cultures. For this, medium was aspirated from all wells and replaced with 50 µl of a 1:1 mixture of CellTiter Glo3D reagent and Hanks' Balanced Salt Solution (HBSS, Gibco #14,025,092). The plate was then shaken for 15 min at 300 rpm (1 mm displacement) to lyse the cells and initiate the light reaction. 24 µl of the lysate was transferred to a white 384-well plate and luminescence was recorded. Luminescence was measured from lysate in graft chambers and perfusion lanes separately. Relative luminescence units were normalized to untreated condition which was defined 100% viable.

#### Azathioprine exposure

After 5 days of co-culture, vascularized spheroid co-cultures were exposed to 50 µM Azathioprine (Sigma-Aldrich, PHR1282) or to vehicle control (0.1% DMSO). 50 µl of 50 µM Azathioprine or vehicle control was added to all perfusion channels in-and outlets (in MV2 medium) and to the graft chamber (in HPM medium), and the plates were placed back in the incubator on an interval rocker switching between a + 14° and -14° inclination every 8 min (OrganoFlow S, Mimetas). After 48 h, medium was collected for LDH quantification, and the perfusion assay was performed. Subsequently, medium was removed, and 5 µM of SytoX™ Green Nucleic Acid Stain (Thermo Fisher Scientific, #S7020) in MV2 was added to all perfusion channel in- and outlets and to the graft chamber for 15 min. Dead cells were visualized using a ImageXpress XLS Micro High content imaging system at 37 °C (4 × and 10 × objective). Dead nuclei quantification was performed using FIJI v.1.52 by thresholding cropped images (removing the lateral perfusion channels) using the “default” algorithm followed by “watershed” and “analyze particles.” LDH release was performed using LDH™-Glo cytotoxicity assay (Promega, #J2380, sample dilution 1:75) according to the manufacturer’s instructions.

#### Statistical analysis

Statistical analysis was done in GraphPad Prism 8 (GraphPad Software, USA), and statistical significance was attributed to values of *P* < 0.05 as determined by Student’s *t* test or two-way ANOVA analysis, as described in the figure legends. Data are expressed as mean ± SD or SEM as described in the figure legends.

## Results

### The OrganoPlate Graft allows for the generation of robust microvascular beds

The OrganoPlate Graft consists of 64 microfluidic chips patterned underneath a standard 384-well plate (Fig. 1a). Each chip comprises two perfusion channels and a gel channel, which are connecting six wells per chip (Fig. 1a) The perfusion channels and gel channel join in a central chamber, called the graft chamber (Fig. 1b). The graft chamber has an opening at the top that allows placement of a target tissue. Two phaseguides [40, 41] on the bottom surface of the microfluidic layer allow filling the graft chamber with an extracellular matrix (ECM) gel. Phaseguides are small ridges at the bottom of a microfluidic channel that act as capillary pressure barriers for an advancing liquid–air meniscus. In this manner, a liquid ECM gel precursor can be added to the graft chamber without overflowing into the adjacent perfusion channels. The use of surface tension techniques for ECM patterning obviates the need for artificial membranes in creating a layered culture setup [41, 42]. In this study, we exclusively utilized collagen I to pattern the microfluidics; however, several different ECM gels have been reported in conjunction with phaseguide technology [43–45]. The process of in vitro tissue grafting consist of four main steps, as illustrated in Fig. 1b: First, a collagen-I gel precursor is dispensed into the gel inlet (A2 in Fig. 1b) with subsequent gelation for 15 min at 37 °C. Next, RFP-expressing human HUVECs are introduced in the lateral perfusion channels (inlets A1 and A3 in Fig. 1b). Once cells settled and attached to the bottom of the channel, gravity-driven perfusion was initiated by placing the plate on an interval rocker (Supplementary Fig. 1a). After three days of culture, two confluent endothelial tubules (here called lateral vessels) were formed in the perfusion channels with lumen that are accessible from the in- and outlets (A1, B2, and A3, B3 in Fig. 1b). As a third step, a cocktail of four angiogenic factors is added to the graft chamber (B2 in Fig. 1b). The combination of VEGF, S1P, PMA, and bFGF induced sprouting of the endothelial vessels into the adjacent collagen-I gel with a tip-stalk cell hierarchy, typical for the angiogenesis process [46]. After four days of exposure to the angiogenic factors, a microvascular bed was formed with microvessels spanning the length from the lateral vessels to the graft chamber opening (Fig. 1c and Supplementary Video 1). As a last step, angiogenic factors are removed and a target tissue is placed in the graft chamber on top of the microvascular bed (Fig. 1c). Figure 1d shows a microvascular bed that was stained for the endothelial marker CD31 in combination with a nuclear stain, including a reconstruction of a cross section orthogonal (*y*–*z*) and parallel (*x*–*z*) to the sprouting direction. Cross sections reveal that microvessels have a lumen and penetrate the collagen toward the opening of the graft chamber. To characterize the reproducibility of vascular bed formation between chips and plates, we assessed variation of 64 vascular beds in three independent plates (Supplementary Fig. 1b, c). Coverage of the microvascular bed area as a percentage of the total surface area showed low chip-to-chip and plate-to-plate variations (Fig. 1e) with average coverage of 18.4 ± 2.2% (SD). No difference was observed between the three plates. Sprouting of microvessels toward the graft chamber occurred radially toward the graft chamber opening, with 40.19 ± 1.6% (SD) of structures falling between 80.5° and 100° (Fig. 1f). Orientation was defined with respect to the longitudinal direction of the phaseguide and lateral perfusion channels. Upon removal of angiogenic factors, pre-formed hepatocyte spheroids (Supplementary Fig. 2a) were transferred from low attachment plates to the graft chamber (Fig. 1c). The spheroids were placed on top of the microvascular bed either manually or using a pipetting robot (Supplementary Fig. 2b and c and Supplementary Video 3). Robot-operated tissue placement provided better centering of the tissue compared to manual transfer (Fig. 1g) with an average displacement of 131 ± 56.2 µm (SD) with respect to the center of the inlet compared to manual positioning displacement of 198 ± 59.2 µm (SD).

### Vascular remodeling is induced by hepatic microtissues

**Fig. 2:**
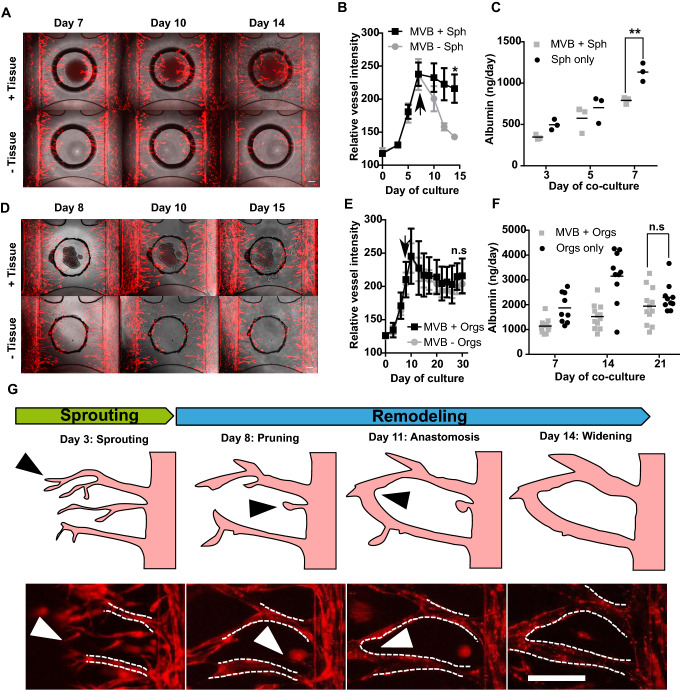
Vascular remodeling is induced by hepatic microtissues. **a** Phase-contrast images with fluorescence overlay of hepatocyte spheroids grafted on top of a microvascular bed (top panel) in comparison to a microvascular bed only (bottom panel) at day 7, 10 and 14 of culture which represent day 0, 3, and 7 of co-culture. Scale bar: 200 µm. **b** Relative microvascular bed (MVB) RFP signal in presence (black squares) or absence (gray circles) of hepatocyte spheroids (Sph) during 14 days of culture. Spheroids were transferred at day 7 of culture as indicated by the black arrow. Data represents mean ± SD, *n* = 8–16, statistical significance was attributed to value of *P < 0.01 as determined by unpaired Student’s *t test* on day 14 timepoint. **c** Albumin secretion of hepatocyte spheroids during 7 days of co-culture in the presence (gray squares) or absence (black circles) of a microvascular bed. Dots represent individual chips, line represents mean, *n* = 3, statistical significance was attributed to value of **P < 0.001 as determined by unpaired Student’s *t* test on day 14 timepoint. **d** Phase-contrast images with fluorescence overlay of hepatic organoids grafted on top of a microvascular bed (top panel) in comparison to a microvascular bed only (bottom panel) at day 8, 10 and 14 of culture which represent day 0, 2 and 6 of co-culture. Sale bar: 200 µm. **e** Relative microvascular bed (MVB) RFP signal in the presence (black squares) or absence (gray circles) of hepatic organoids (orgs) during 30 days of culture. Organoids were transferred at day 8 of culture as indicated by the black arrow. Data represent mean ± SD, *n* = 8–14, significance was calculated by unpaired Student’s *t* test on day 30 timepoint and shown as non-significant (n.s., *P* > 0.05). **f** Albumin secretion of hepatocyte organoids during 21 days of co-culture in the presence (gray squares) or absence (black circles) of a microvascular bed. Dots represent individual chips, line represents mean, *n* = 5–9, significance was calculated by unpaired Student’s *t test* on day 21 timepoint and shown as non-significant (n.s., P > 0.05). **g** Angiogenesis process of RFP-labeled HUVEC during initial sprouting and subsequent vascular structures remodeling in the presence of a hepatocyte spheroid from day 7 of culture. Pictures show filopodia (left, white arrow), microvessel retraction (middle left, white arrow), anastomosis (middle right, white arrow) and widening (right). Scale bar: 200 µm

The interaction of a hepatic spheroid with the pre-formed microvascular bed was assessed. Microvascular beds were formed from RFP-HUVECs, as described before. After four days of angiogenesis, the sprouting cocktail in the graft chamber was replaced with HPM. Lateral perfusion channels were supplied with MV2 medium. Hepatocyte spheroids comprising of 20,000 hepatocytes were placed in the graft chamber, on top of the collagen matrix hosting the microvascular bed and the co-culture was maintained for 7 days (Supplementary Video 2) with medium changes every 2–3 days. Figure 2a shows images of the vascular bed and Fig. 2b the quantification of the area covered by RFP-HUVEC vascular bed in the graft chamber with and without the addition of a hepatocyte spheroid upon removal of angiogenesis factors at day 7. In the presence of a spheroid, microvasculature was maintained over the co-culture period of 7 days, whereas in its absence, the microvascular bed underwent pruning, resulting in an almost complete retraction of the microvessels. Interestingly, when no sprouting cocktail was used, the presence of the spheroid alone was sufficient for inducing sprouting after 7 days of co-culture (Supplementary Fig. 3a and b). These results indicate that hepatocyte spheroids possess high angiogenic potential and support existing microvasculature. To confirm maintenance of hepatic function during co-culture, we assessed release of albumin. Figure 2c shows a similar trend in albumin secretion over time for spheroids in the presence or absence of a vascular bed. However, we observed a generally lower albumin release when spheroids were co-cultured with microvessels. Still, we can conclude that hepatic function was maintained in a vascularized context over the period of co-culture. Co-culture with spheroids also appears to increase barrier integrity of the lateral vessels, where we observed reduced FITC-dextran leakage in the graft chamber opening and the surrounding gel (Supplementary Fig. 4). We then investigated hepatic organoids as an alternative hepatocyte source. Hepatic organoids have been established by Hu et al. using mouse and primary human hepatocytes [47]. These organoids have been reported to allow expansion for multiple months while maintaining key morphological features and hepatic functionalities. Moreover, these human hepatocyte organoids maintain remarkable regenerative capacity, as shown by extensive proliferation after engraftment into mice [47]. Here, human hepatic organoid aggregates of uniform size and shape (Supplementary Fig. 5a) were transferred to the OrganoPlate Graft on top of pre-formed microvascular beds. In contrast to hepatocyte spheroids, which were directly exposed to the medium in the graft chamber, hepatocyte organoids were additionally overlayed with a Matrigel droplet (Supplementary Fig. 5b). Upon tissue placement, medium in the graft chamber was replaced with Hep-medium while MV2 medium was kept in the perfusion channels. Figure 2d shows images of the vascular bed with and without grafted organoids, Fig. 2e quantification of the vascular bed area, and Fig. 2f albumin release over the culture period of 30 days (corresponding to 21 days of co-culture). Similar as for hepatocyte spheroids, microvasculature integrity was maintained when co-cultured with hepatic organoids (Fig. 2d). However, we did not observe a regression of the vasculature in absence of the organoids, possibly because the growth factors in Hep-medium were sufficient for stabilizing the microvasculature (Fig. 2e). To confirm this, we cultured microvascular beds in basal Hep-medium in the graft chamber supplemented with either HGF, FGF10 or EGF at concentrations previously described [44] and we observed that HGF alone was capable of stabilizing the vascular structures over a period of 6 days (Supplementary Fig. 6). Similar to hepatocyte spheroids, barrier integrity of the lateral vessels appeared to be increased when organoids were co-cultured with microvessels (Supplementary Fig. 4). A hepatic phenotype was maintained during the culture period, as indicated by albumin release (Fig. 2f), expression of hepatocyte nuclear factor 4 alpha (HNF4α), acetylated tubulin (Ac. Tub) and E-cadherin (ECAD) (Supplementary Fig. 5c). Furthermore, hepatic organoids incubated with the fluorescent compound 5-chloromethylfluorescein diacetate (CMFDA) showed functionality and polarization of the canalicular MRP2 transporter (Supplementary Fig. 5d) and were capable of producing and secreting urea throughout the 21 days of co-culture (Supplementary Fig. 5e).

During the vascularization process of hepatocyte spheroids, the endothelial vessels undergo all stages typical of the angiogenesis process (Fig. 2g). HUVECs migrate and elongate toward the graft chamber with a clear presence of filopodia in tip cells leading the emerging microvessels (Fig. 2g, left). Upon removal of angiogenic factors and placement of the hepatic spheroids, non-connected sprouts undergo pruning (Fig. 2g, middle left), whereas others undergo anastomosis (Fig. 2g, middle right) and show overall widening of microvascular structures (Fig. 2g, right). As a result of this extensive remodeling, a vascular structure emerges with distinct, spatially separated afferent vessels that developed orthogonally from the lateral vessels toward the hepatic spheroids.

### Endothelial cells penetrate hepatic microtissues forming lumenized, perfusable microvasculature

**Fig. 3:**
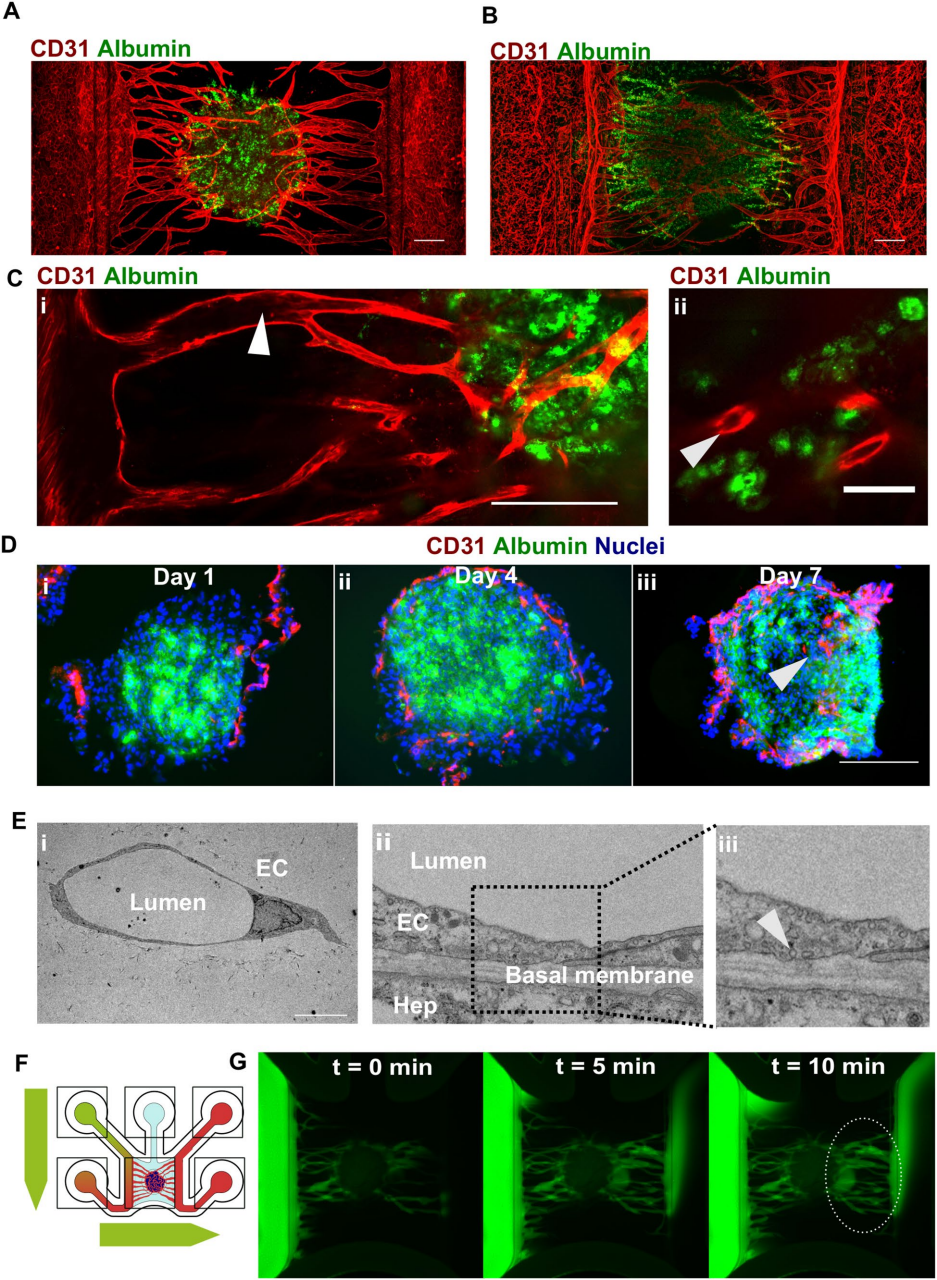
Endothelial cells penetrate hepatic microtissues forming lunemized, perfusable microvasculature. **a** Maximum intensity projection of hepatocyte spheroid co-cultured with microvessels for 7 days and stained against albumin (green) and CD31 (red). Scale bar: 200 µm. **b** Maximum intensity projection of hepatocyte organoids co-cultured with microvessels for 21 days and stained against albumin (green) and CD31 (red). Scale bar: 200 µm. **c** Single-plane confocal images of hepatocyte spheroid co-cultured with microvessels for 7 days and stained against albumin (green) and CD31 (red). Lumenized spaces are observable in the vessels connecting the main tubes to the hepatocyte spheroid (I, white arrow) and in close proximity of hepatocytes (ii, white arrow). Scale bar: 200 µm (i) and 50 µm (ii). **d** Cryo-sections of spheroids co-cultured for 1 (i), 4 (ii), and 7 (iii) days on microvascular beds stained against albumin (green), CD31 (red), and nuclei (blue). White arrow indicates penetration of CD31 + endothelial cells inside the hepatocyte spheroid. Scale bar: 200 µm. **e** EM images of sections of hepatocyte organoids co-cultured with microvessels for 7 days indicating endothelial cells (EC) with distinct lumen (i) and vascular-hepatic contact point (ii) with intracellular pinocytic vesicles (iii, white arrow). Scale bar: 10 µm. **f** Schematic depiction of flow through the chip to assess perfusability of vascularized liver cultures using 150-KDa FITC-labeled dextran. **g** Over-time fluorescence microscopy images of 150-KDa FITC-labeled dextran perfusing a hepatocyte spheroid co-cultured for 7 days with microvessels. Dotted ellipse indicates FITC-labeled dextran emerging from the spheroid and flowing in the lateral perfusion channel

Figure 3a, b respectively shows vascularized spheroids and organoids resulting from 7 days (spheroids) and 21 days (organoids) of co-culture with microvessels stained for CD31 and albumin. Confocal imaging revealed the presence of lumenized spaces in the afferent vessels connecting the spheroid with the lateral vessels (Fig. 3c, i) and in smaller vessels in close proximity to hepatocytes (Fig. 3c, ii, Supplementary Fig. 3c). We observed the presence of microvascular structures at different depths throughout the hepatic spheroids (Supplementary Fig. 7a and Supplementary Fig. 7b). To further validate whether microvascular remodeling leads to penetration of microvessels in the hepatic microtissues, hepatocyte spheroids were co-cultured with microvascular beds and extracted after 1, 4 or 7 days followed by cryosectioning and immunostaining for CD31 and Albumin. At day 4, endothelial cells appeared to superficially interact with hepatocyte spheroids (Fig. 3d, ii). At day 7, however, endothelial cells penetrated the hepatocyte spheroids (Fig. 3d, iii). Vessel lumen was confirmed by TEM imaging (Fig. 3e, i) which also revealed that vessels in direct contact with hepatic organoids showed the presence of pinocytic vesicles, suggesting transport through the vascular wall as well as ECM deposition (Fig. 3e, ii). Next, we assessed whether the vascular network could be perfused with a 150 kDa FITC-labeled dextran. A hydrostatic pressure difference was applied between the inlets connecting the left and right lateral vessels, forcing the fluorescent molecules through the microvascular bed (Fig. 3f and Supplementary Fig. 7c). Indeed, it was possible to observe a flux of fluorescent dye from one lateral vessel through the microvascular network and the spheroid into the other lateral vessel on the opposite side (Fig. 3g). For organoids, we were not able to observe complete interconnection (data not shown). Despite not detrimental for vascular structures, hepatic organoids in this context were not capable of significantly promoting further vascular structures remodeling as hepatic spheroids were.

### Modeling veno-occlusive disease

**Fig. 4:**
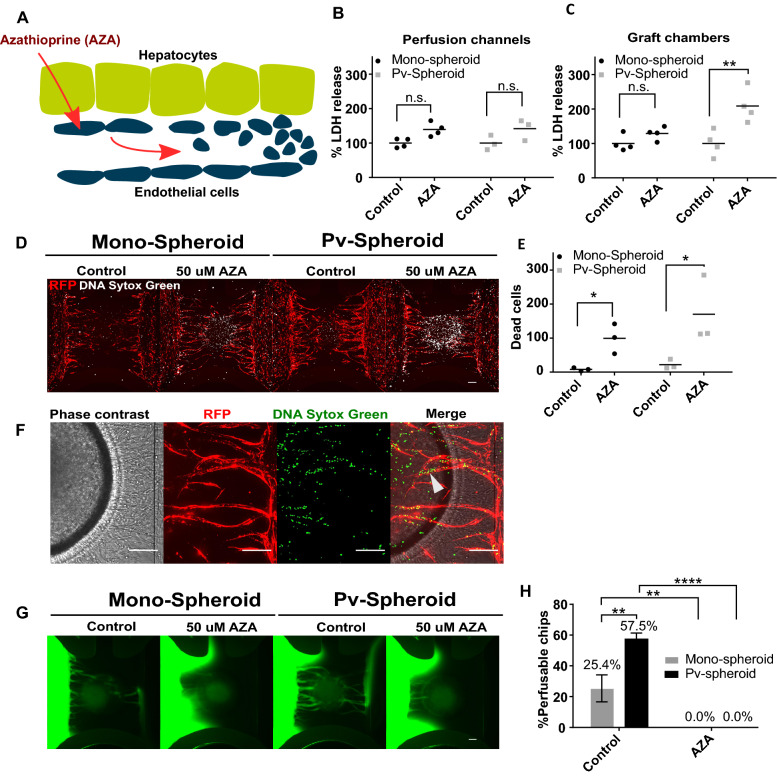
Vascularized human liver model of toxin-induced veno-occlusive disease. **a** A schematic diagram of Azathioprine-induced hepatic veno-occlusive disease. **b** Perfusion lane LDH release upon exposure to 50 µM azathioprine (AZA) for 48 h (days 5–7 of co-culture with microvessels). Dots represent individual chips, line represents mean, *n* = 3–4, significance was calculated using two-way ANOVA and shown as non-significant (n.s., Tukey’s multiple comparison test, *P* > 0.05). **c** Graft chamber LDH release upon exposure of 50 µM azathioprine for 48 h (days 5–7 of co-culture with microvessels). Dots represent individual chips, line represents mean, *n* = 3–4. Significance was calculated using two-way ANOVA (Tueky’s multiple comparison test, n.s, *P* > 0.05, **P < 0.01) **d** Fluorescent images overlay of RFP-HUVEC (red) with dead cells (white) after 48 h exposure to 50 µM AZA. **e** Quantification of dead cells in the graft chamber upon exposure to 50 µM AZA. Dots represent individual chips, line represents mean, *n* = 3, significance was calculated using two-way ANOVA (Tukey’s multiple comparison test, *P < 0.05). **f** representative images of pre-vascularized spheroid cultures exposed to 50 µM AZA indicating the presence of dead cells inside intact vessels (white arrow). Scale bar = 200 µm. **g** Representative fluorescent images of FITC-labeled dextran after 5 min of perfusion. Dotted ellipse indicates FITC-labeled dextran emerging from the spheroid and flowing in the lateral perfusion channel. **h** Percentage of perfusable chips/OrganoPlate (8 chips/condition in a total of five plates) after AZA treatment. Bar represents mean ± SEM, *n* = 5. Significance was calculated using two-way ANOVA (Tukey’s multiple comparison test, *P < 0.05, ****P < 0.0001)

Azathioprine (AZA) is an immunosuppressive agent used in organ transplantation to prevent rejections. It is associated with veno-occlusive disease (VOD), also known as sinusoidal obstruction syndrome (SOS). Its proposed mechanism of toxicity involves glutathione depletion in liver endothelial cells [37], followed by apoptosis of these cells and their extrusion into sinusoids, leading to obstruction and congestion and subsequent reduction of blood flow [48]. Therefore, it was investigated whether a vascularized liver spheroid model could capture congestion and loss of perfusability upon administration of Azathioprine (Fig. 4a). We first assessed whether AZA appears to be toxic to the culture as a whole. For this, we exposed vascularized and non-vascularized hepatocyte spheroids to a dose range of AZA and we assessed viability using a commercially available intracellular ATP readout. We measured ATP release in the graft chamber or in the perfusion lanes (Supplementary Fig. 8a). This allowed us to partially discriminate the toxic effect on the hepatocytes and lateral endothelial cells. Based on this initial result, we selected a concentration of 50 µM and an exposure time of 48 h which we did not expect to result in a substantial loss of viability of the culture. Next, we tested two types of spheroids: spheroids comprising hepatocytes only (mono-spheroids) and pre-vascularized spheroids (pv-spheroids) comprising hepatocytes and RFP-HUVECs in a 20:1 ratio. We observed the formation of an intricate endothelial network inside the pv-spheroids after 2 days of aggregation (Supplementary Fig. 9a). Mono-spheroids and pv-spheroids were cultured with microvessels for 5 days, where we observed a larger extent of endothelial structures within the pv-spheroids (Supplementary Fig. 9b). Afterward, the cultures were exposed to 50 µM AZA for 48 h. To assess the effect of AZA on the culture as a whole, we opted for administering the drug to both, perfusion channels and graft chambers. To assess toxicity, we measured lactate dehydrogenase (LDH) released in medium samples from both the graft chamber and the perfusion channels. No significant increase in secreted LDH was observed in the perfusion channels (Fig. 4b, Supplementary Fig. 8b), indicating that the lateral vessels could tolerate exposure to AZA. LDH release in the graft chamber showed a significant increase, but only for pv-spheroids (Fig. 4c). AZA exposure did not result in evident morphological changes of the microvessels and spheroids for either mono-spheroids or pv-spheroids (Supplementary Fig. 9c). Next, cultures were stained with Sytox Green to visualize dead cells. A substantial number of dead cells was observed in the graft chamber due to AZA exposure, particularly at the regions of hepatic-endothelial interactions (Fig. 4d, e). Higher magnification confocal imaging revealed the presence of dead cells in the afferent vessels and the microvasculature in proximity to the hepatocyte spheroids (Fig. 4f). To assess whether congestion of microvasculature with dead cells caused impaired perfusion as observed during VOD, perfusability of the two types of spheroid cultures was assessed. FITC-labeled dextran was added to one lateral vessel, and perfusion toward the second lateral vessel was observed (Fig. 4g). Mono-spheroid and pv-spheroid cultures were perfusable in 25.4% and 57.5% of the cases, respectively, whereas none of the AZA-exposed cultures was perfusable (Fig. 4h). Taken together, these results demonstrate that congestion of microvasculature due to AZA exposure could be recapitulated in a vascularized liver model and that FITC-dextran perfusability could be utilized as a functional readout.

## Discussion

In vitro grafting of tissues is at the pinnacle of cell culture, adding an element that otherwise could only be achieved in vivo, i.e., through xenografting techniques. Microfluidic cell culture techniques are ideally suited for providing the perfusable microvascular network, and the open-top channel approach makes these amenable to receiving a donor tissue, the dimensions of which may well exceed those of the microfluidic channel. In this study, we demonstrated clear feasibility of in vitro grafting by example of vascularization of liver tissue. We demonstrated that the liver construct could be fluidically connected to the microfluidic vascular bed and subsequently perfused. Moreover, we showed conceptual application of the vascularized system by mimicking veno-occlusive disease and providing loss of perfusability as a phenotypic readout. We propose, therefore, a viable, robust, easy-to-use, and versatile platform to develop vascularized in vitro cultures. In this study, we focused on liver as a testbed for demonstrating the handling and capabilities of the platform. While more investigation is needed to fully capture the effect of vascularization on hepatic function, we were able to assess and confirm a functional hepatic population in a vascularized context. We further envision flow-cytometry as a future direction for further hepatic characterization.

In this study, we used three types of liver tissue constructs: liver spheroids, pre-vascularized liver spheroids, and liver organoids. Vascular beds in the presence of both liver spheroids and pre-vascularized spheroids underwent clear anastomosis. However, engraftment of pre-vascularized hepatic spheroids yielded a twofold higher success rate of forming a culture that could be perfused completely. We can, thus, conclude that constructs with endogeneous endothelium are more likely to produce a functional network, a strategy that has also proven successful in vivo [49, 50]. Liver organoids did not convincingly induce anastomosis or subsequent perfusability. We suspect that the composition of the growth factor-rich medium used for organoids is the primary cause for this as many Hep-medium components (HGF, EGF, FGF7, and FGF10) are known to support or induce angiogenesis [51–54]. Indeed, HGF alone was capable of preventing regression of microvasculature in the absence of hepatic organoids. Additionally, we suspect that the existence of a collagen–Matrigel interface might hinder further vasculature elongation, thus, preventing complete interconnection. Further study would include reduction in growth factors in the Hep-medium to promote local gradients, as well as investigating alternatives to Matrigel which might pose a significant hindrance for vascular development.

We mimicked induction of VOD by exposure to azathioprine. While having a marginal toxic effect on the culture as a whole, azathioprine had a profound effect on the perfusability of the network. In addition, higher magnification confocal imaging revealed the presence of dead cells in the afferent vessels and the microvasculature in proximity to the hepatic spheroids. Interestingly, while LDH was not substantially increased in mono-spheroids, we did observe graft chamber elevation after treatment for cultures with pre-vascularized spheroids. This suggests that cell death did occur but predominantly in locations with dense microvascular structures.

For this study, we used HUVECs to form the vascular bed, study interaction with the target tissue, and model disease. HUVECs are a convenient source of endothelial cells that are widely available, form robust tubes, and undergo reproducible angiogenesis in our platform. Future work will focus on incorporating Liver sinusoidal endothelium (LSECs) which is a highly specialized form of endothelium that is, among other things, characterized by fenestrae. These small openings act as a selective “sieve” and allow for bi-directional exchange of molecules between sinusoidal blood and liver parenchyma [55]. Inclusion of LSECs could be done by either growing them as a vascular bed or incorporating them with the liver spheroid and connecting them to the host vascular bed. In addition, we foresee the incorporation of other non-parenchymal cells, including stellate and Kupffer cells. We expect that such co-cultures aid long-term stability and metabolic competence of the in vitro culture and allow studying disease conditions such as liver fibrosis, steatosis, and possibly even using the model to assess liver infection by, for example, Hepatitis B-Virus and malaria-causing parasites.

We investigated the grafting of spheroids composed of cells from an immortalized cell line and organoids derived from liver stem cells. Another source of liver material would be tissue slices from donor livers. In that case, the tissue includes endogenous endothelium that could be used to study its ability to connect to the host vascular bed when in a sufficiently viable state. Another route to study liver vascularization is to use stem cell-derived organoids that undergo the various stages of embryonic development [56]. Such organoids are typically known to comprise all parenchymal and non-parenchymal cells present in the liver. In this case, it would be highly interesting to study the connection of endogenous endothelium with the host vascular bed. We anticipate that the timing of the grafting step and tuning of growth factors will prove crucial for effective co-development of the liver organoid and its vascular structure.

Although we focused on the liver as a proof-of-concept organ in this publication, the platform is, in essence, applicable to the vascularization of any tissue. We anticipate that such added vasculature will become crucial in the rapidly developing organoid field, potentially leading to, e.g., blood–brain barrier type endothelium in brain organoids [57], perfused glomeruli in kidney organoids [58, 59], and functional beta islets for pancreatic organoids [60].

The platform also holds promise to vascularize tumor tissue. Today tumor vascularization is typically studied in patient-derived xenografts (PDX), where tumor explants are implanted in immunodeficient or humanized mice. These models are typically exposed to therapeutic compounds to study their efficacy on a personalized level. PDX models are valuable due to their intrinsically complex microenvironment. However, their establishment is very time consuming, cumbersome, and with variable success rates. Therefore, such PDX studies are primarily used for retrospective rather than prospective studies [61, 62]. An in vitro grafting protocol holds the promise of being faster, more reproducible, more cost effective as well as easier to analyze than classical PDX approaches. Alternatively, this platform could be used to generate more relevant PDX explant cultures currently used in drug and biomarker discovery. Grafted PDX explant cultures would eventually allow for the inclusion of relevant immune cells, overcoming in part a limitation of using PDX models in immune oncology [63–65].

Until today, the study of tissue vascularization is limited to laboratories that have access to animal testing facilities. The platform demonstrated here shows clear proof-of-concept of vascularization of tissues in vitro. Not only was the concept of vascularizing in vitro proven feasible, but the platform also allows routine experimentation with sufficient throughput, fully compatible with (automated) microscopes and robotic handling. The accessibility of the platform for any laboratory around the world and its usability by any cell culture biologist will provide a great push forward toward routine inclusion of perfused vasculature in tissue culture protocols.

## Supplementary Information

Below is the link to the electronic supplementary material.

Supplementary Fig. 1: Reproducibility of microvascular bed formation. a Schematic representation of the passive pumping method used to perfuse lateral tubes and microvessels. Every 8 min, the rocker platform (α = 14° angle) switches from theleft (a-b) to the right (c-d) allowing bidirectional perfusion. b Generation of 64 identical microvascular beds after 4 days of sprouting using RFP-labelled HUVECs. Scale bars: 200 µm. c Schematic showing the automated image analysis steps used to quantify microvessels area and orientation in the OrganoPlate Graft. After acquisition of confocal images, the region of interest (ROI) corresponding to the graft chamber is selected and pictures are converted into color coded and binary data and analyzed for microvessels orientation and area, respectively. Supplementary file 1 (EPS 109,638 kb)

Supplementary Fig2: Microtissue formation and placement in the OrganoPlate Graft. a Aggregation of UHH in ultra-low attachment 96-well plate for 24 hours. Scale bars : 300 µm. b Phase-contrast images of 64 spheroids positioned in the graft chamber of the OrganoPlate Graft either manually (left) or using a liquid handling robot (right). Scale bars: 200 µm. c Schematic of microtissue placement protocol. Spheroids or organoids are picked up from ultra-low attachment 96-well plate (step 1-2) and transferred on top of the microvascular bed in the OrganoPlate Graft (step 3-5) using wide-bore pipette tips. Supplementary file 2 (EPS 75,066 kb)

Supplementary Fig. 3: Spheroids induce spontaneous sprouting. a Images of hepatocyte spheroids culture on non-sprouted lateral RFP-HUVEC tubes showing spontaneous sprouting after 7 days of co-culture. Scale bars: 200 µm. b Maximum intensity projection of hepatocyte spheroid-induced sprouting after 7 days of co-culture stained against CD31 (red) and albumin (green). Scale bars: 200 µm. c Maximum intensity projection of vascularized hepatocyte spheroid stained against CD31 (red) and albumin (green), dotted square indicates the location of the inlet in Fig. 3C ii). Supplementary file 3 (EPS 6449 kb)

Supplementary Fig. 4: Barrier integrity assay in OrganoPlate Graft. Representative fluorescent images upon perfusion of lateral vessels with FITC-labelled dextran for 8 minutes in presence or absence of hepatic organoids (left panel) after 21 days of co-culture and in presence or absence of hepatocyte spheroids (right panel) after 7 days of co-culture. Circles indicate dye leaking in the graft chamber opening or in the surrounding gel. Supplementary file 4 (EPS 1858 kb)

Supplementary Fig5: Characterization of hepatic organoids in OrganoPlate Graft. a Aggregation of fetal hepatic organoids in ultra-low attachment 96 well plate for 72 hours. Scale bars: 300 µm. b Steps used to generate a microvascular bed with Matrigel embedding. Step 1: The graft chamber is filled with collagen I gel. Step 2: Endothelial cells are seeded in the perfusion channels. Step 3: After lateral vessel formation, angiogenic factor are added on top of the graft chamber to induce microvessels sprouting. Step 4: Once microvessels reached the middle of the ECM gel, organoids are added on top of the microvascular bed and are embedded in a drop of Matrigel. c Immunostaining images showing expression of hepatic markers (albumin, MRP2, HNF4a), polarization marker (Ac. Tub) and epithelial marker (ECAD) in hepatic organoids co-cultured with microvessels. Scale bars: 50 µm. d Confocal image (left) of CFDA-SE indicating bile canaliculi presence in an outgrowth from an organoid co-culture with microvessels. Arrow in the zoom-in picture (right) highlights the presence of canaliculi structures. Scale bar: 200 µm. e Urea secretion of hepatocyte organoids during 21 days of co-culture in presence or absence of a microvascular bed. Dots represent individual chips, line represents mean, n = 3-7. Supplementary file 5 (EPS 3692 kb)

Supplementary Fig. 6: Stabilization of vasculature by growth factors. Relative MVB RFP signal in the graft chamber after 6 days of culture in HHPM or in basal Hep-Medium with the addition of either HGF, FGF-10 or EGF. Data represents mean ± SD, n = 8. Significance was calculated using two-way ANOVA (Tukey’s multiple comparison test, ****P < 0.0001). Supplementary file 6 (EPS 81 kb)

Supplementary Fig. 7: Vascular structures penetrate spheroids. a Maximum intensity projection of hepatocyte spheroid co-cultured with microvessels for 7 days and stained against albumin (green) and CD31 (red). Scale bar: 100 µm. b Single plane confocal images of the same spheroid at different depths. 0 µm indicates bottom region of the spheroid. c Wide-field fluorescent image showing FITC-dextran perfusing through interconnected vessels. d Single plane confocal images showing microvessels (red, CD31) in close proximity and around hepatocytes (green, albumin). White arrow indicate apparent lumenized structures. Supplementary file 7 (EPS 2783 kb)

Supplementary Fig. 8: Viability assessment after AZA exposure. a Viability of spheroids exposed to dose-range of AZA for 72h. Cultures were generated with (black dots) or without (white) pre-grown vascular beds. Left panel shows graft chamber viability percentage as compared to untreated control. Right panel shows perfusion lanes viability percentage as compared to untreated control. Data represents mean ± SD, n = 3. b Graft chamber LDH release upon exposure of 50 µM azathioprine for 48 hours on several culture combinations: Pv-spheroids or mono-spheroids without vascular beds, pv-spheroids or mono-spheroids on unsprouted lateral tubes, vascular beds only, pv-spheroids or mono-spheroids on sprouted vascular beds. Bars represent mean ± SD, n = 3-6. Significance was calculated using two-way ANOVA (Tueky’s multiple comparison test, n.s, P >0.05, *P < 0.01). Supplementary file 8 (EPS 125 kb)

Supplementary Fig. 9: Pre-vascularized spheroids morphology. a Single plane confocal images (left) and maximum intensity projection (right) of a hepatocyte spheroid containing 20,000 UHH and 1000 HUVECs after 2 days of aggregation. b Representative fluorescent overlay images of mono-spheroids and pv-spheroids co-cultured with microvessels for 5 days, red color indicates the localization of RFP-HUVECs. c Representative fluorescent overlay images of mono-spheroids and pv-spheroids after 48 hours treatment with 50 µM AZA, red color indicates the localization of RFP-HUVECs. Supplementary file 9 (EPS 6946 kb)

Supplementary Video 1: 3D reconstruction of microvascular bed in OrganoPlate Graft. Animation showing a 3D reconstruction of a microvascular bed stained against CD31 (green) and DNA (blue) showing presence of lumenized lateral vessels and 3D arrangement of microvessels. Supplementary file 10 (AVI 6184 kb)

Supplementary Video 2: Vascularization process in OrganoPlate Graft. Time lapse of RFP-HUVECs sprouting towards the graft chamber opening and subsequent engraftment of hepatocyte spheroid for additional 48 hours of co-culture. Supplementary file 11 (MP4 7664 kb)

Supplementary Video 3: Automatic microtissue transfer to OrganoPlate Graft. Video shows OT-2 robot (Opentrons) transferring microtissue from ultra-low 96 well plates to OrganoPlate Graft. Supplementary file 12 (MP4 134,481 kb)

## Data Availability

All the data supporting the findings of this study are available from the corresponding author on reasonable request.
